# Using Multiple Ontologies to Integrate Complex Biological Data

**DOI:** 10.1002/cfg.498

**Published:** 2005

**Authors:** Mary Shimoyama, Victoria Petri, Dean Pasko, Susan Bromberg, Wenhua Wu, Jiali Chen, Nataliya Nenasheva, Anne Kwitek, Simon Twigger, Howard Jacob

**Affiliations:** 1 Human and Molecular Genetics Center Medical College of Wisconsin Milwaukee WI 53226 USA; 2 Medical College of Wisconsin 8701 Watertown Plank Road Milwaukee WI 53226 USA

## Abstract

The strength of the rat as a model organism lies in its utility in pharmacology, biochemistry and physiology research. Data resulting from such studies is difficult to represent in databases and the creation of user-friendly data mining tools has proved difficult. The Rat Genome Database has developed a comprehensive ontology-based data structure and annotation system to integrate physiological data along with environmental and experimental factors, as well as genetic and genomic information. RGD uses multiple ontologies to integrate complex biological information from the molecular level to the whole organism, and to develop data mining and presentation tools. This approach allows RGD to indicate not only the phenotypes seen in a strain but also the specific values under each diet and atmospheric condition, as well as gender differences. Harnessing the power of ontologies in this way allows the user to gather and filter data in a customized fashion, so that a researcher can retrieve all phenotype readings for which a high hypoxia is a factor. Utilizing the same data structure for expression data, pathways and biological processes, RGD will provide a comprehensive research platform which allows users to investigate the conditions under which biological processes are altered and to elucidate the mechanisms of disease.


**Comparative and Functional Genomics**
*Comp Funct Genom* 2005; **6**: 373–378.
Published online in Wiley InterScience (www.interscience.wiley.com). **DOI:** 10.1002/cfg.498

**Research Article**

### Using multiple ontologies to integrate complex biological data

Mary Shimoyama*, Victoria Petri, Dean Pasko, Susan Bromberg, Wenhua Wu, Jiali Chen, Nataliya Nenasheva, Anne Kwitek, Simon Twigger and Howard Jacob
*Human and Molecular Genetics Center, Medical College of Wisconsin, Milwaukee, WI 53226, USA*

*Correspondence to: Mary Shimoyama, Medical College of Wisconsin, 8701 Watertown Plank Road, Milwaukee, WI 53226, USA. E-mail: mshimoyama@hmgc.mcw.edu*

Received: 12 September 2005
Revised: 17 October 2005
Accepted: 7 November 2005

### Abstract

**The strength of the rat as a model organism lies in its utility in pharmacology, biochemistry and physiology research. Data resulting from such studies is difficult to represent in databases and the creation of user-friendly data mining tools has proved difficult. The Rat Genome Database has developed a comprehensive ontology-based data structure and annotation system to integrate physiological data along with environmental and experimental factors, as well as genetic and genomic information. RGD uses multiple ontologies to integrate complex biological information from the molecular level to the whole organism, and to develop data mining and presentation tools. This approach allows RGD to indicate not only the phenotypes seen in a strain but also the specific values under each diet and atmospheric condition, as well as gender differences. Harnessing the power of ontologies in this way allows the user to gather and filter data in a customized fashion, so that a researcher can retrieve all phenotype readings for which a high hypoxia is a factor. Utilizing the same data structure for expression data, pathways and biological processes, RGD will provide a comprehensive research platform which allows users to investigate the conditions under which biological processes are altered and to elucidate the mechanisms of disease. Copyright © 2006 John Wiley & Sons, Ltd.**

**Keywords:** **RGD; Rat Genome Database; phenotype; ontology; physiology**

### Introduction

Initially, the Rat Genome Database (RGD) used ontologies to provide a simple framework for classifying, representing and navigating across gene, phenotype and disease information to link genomic data to function and disease (Ashburner and Lewis, 2002; Stevens *et al.*, 2000) and as a means of providing a view of biological information in the context of the genome. RGD implemented four ontologies: Gene Ontology (GO), Mammalian Phenotype Ontology (MP), Disease Ontology (DO) and a PathWay ontology (PW). The MP was initially developed at Mouse Genome Informatics (Smith *et al.*, 2005). The disease ontology was adapted from the Medical Subject Headings (MeSH; Nelson *et al.*, 2001) and the pathway ontology was developed at RGD in order to integrate data from existing pathway databases, such as the Kyoto Encyclopedia of Genes and Genomes (Kanehisa, 2002), REACTOME (Joshi-Tope *et al.*, 2005), GenMapDB (Dahlquist *et al.*, 2002) and the Biomolecular Interaction Database (Bader *et al.*, 2003), as well as pathway data found in the literature. It also includes 'altered pathway' terms to allow for the representation of pathways whose events or interactions are altered by genetic or environmental factors.

Ontology annotations were also used in tools at RGD to provide a view of biological information in the context of the genome. Such tools include the GViewer (Figure 1), which provides a genome-wide view of the genes and QTLs related to a single or multiple ontology query, and GBrowse, which provides ontology tracks showing gene function,

Copyright © 2006 John Wiley & Sons, Ltd.

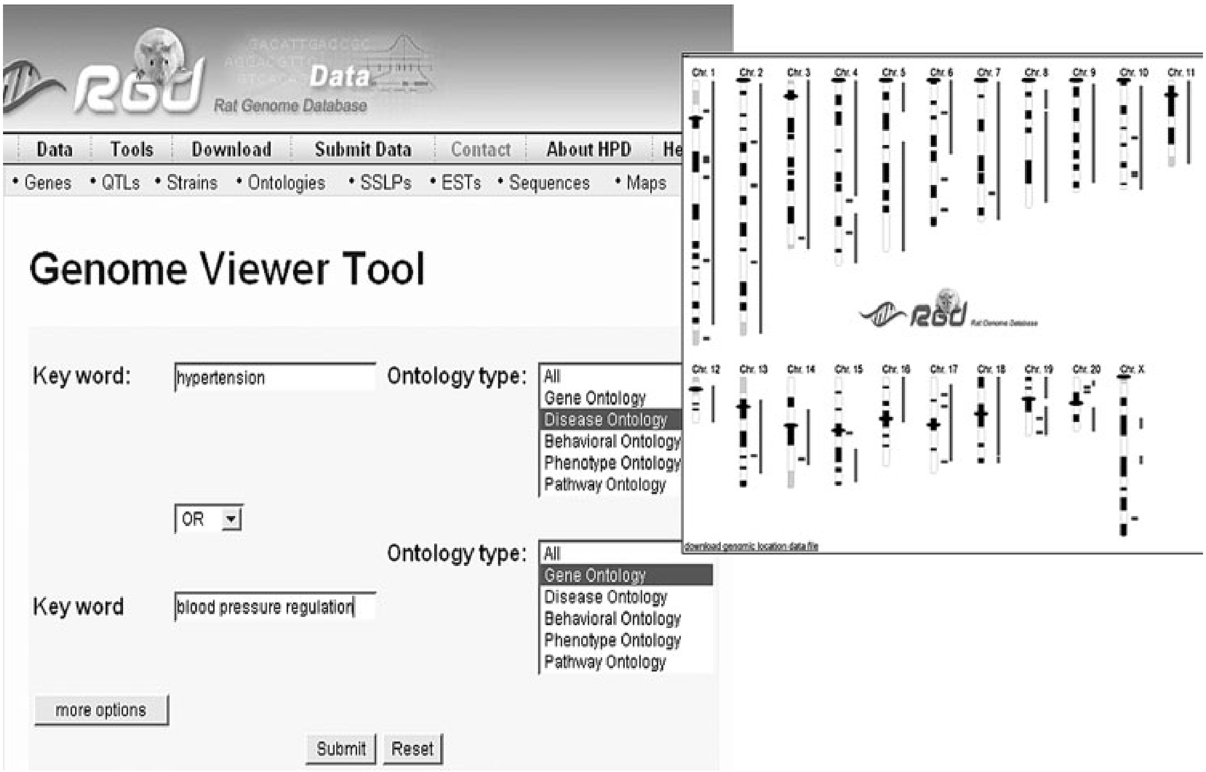

**Figure 1.** Genome viewer tool pathway, disease and phenotype information in the context of the genome.

For the more complex data generated by much of the rat research community, the simple annotations provided by single ontologies are insufficient. They do not answer questions about the conditions under which the biological phenomena take place or what factors could inhibit or modify them. They also do not provide a mechanism for relating disparate types of biological information to allow researchers to elucidate patterns or mechanisms involved in disease. Thus, RGD developed a structure that would allow the integration of multiple ontology annotations, as well as qualifiers and actual values, into a single record.

### Methods

Two major changes were made to the existing ontology data structure at RGD. The first were additional fields added to the primary annotation table (Full_Annot Table in Figure 2). The existing Full_Annot table was modelled after that used by many model organism databases to store GO data. This table included such fields as ontology term, term ID, aspect (or identifier for ontology used), notes, object symbol and ID (referring to genetic or genomic object, such as gene and its associated database ID). To accommodate the information necessary for more complex data, additional fields were added to the Full_Annot table. These included *value* for actual phenotype values, as well as expression values; *duration*, to indicate the time frame of treatments or conditions; *qualifier*, which provides flexibility for adding such information as resistance or susceptibility for disease or induced phenotype terms; and a number of statistics fields.

The next area of change included the creation of two additional tables for assay and protocol information and a unique key consisting of the term key, reference ID, evidence code, RGD ID and experiment ID. The relationships among multiple ontology annotations and values for a single record are achieved through an Experiment/Assay table (situated below the Full_Annot table in Figure 2).

Copyright © 2006 John Wiley & Sons, Ltd.

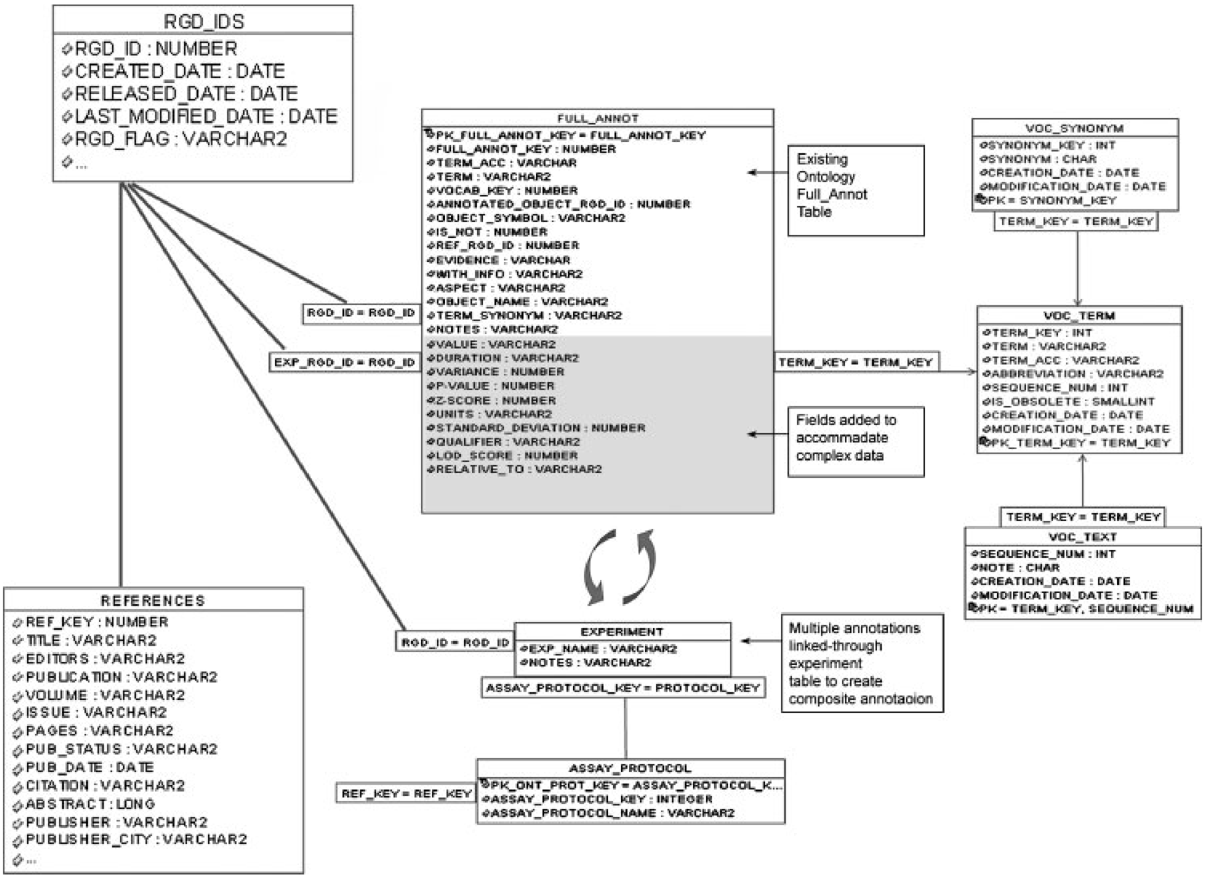

**Figure 2.** Table structure for multiple ontogenies

Through this table, an annotation incorporating a MP ontology term (decreased eosinophil count) and ID, value, evidence code, and reference can be linked to an annotation with an Environmental Factor Ontology term for diet (decreased sodium content), value and units, and these can be linked to another annotation including an Environmental Factor Ontology term for atmosphere (decreased oxygen), value and units, and finally to a fourth annotation including a Genetic Factor Ontology term (female) to create a composite annotation indicating that, for the LE/BluGill strain, a decreased eosinophil count (0.0089E3) was found under the conditions of a decreased sodium diet (0.4%) and decreased oxygen (12%) in females (Figure 3).

Such a composite annotation can be associated with the genomic and genetic elements in RGD, such as genes or mapped phenotypes (Quantitative Trait Loci) or as in this case, a strain. A composite annotation can also stand alone as an experimental record relating phenotypes, drugs, diseases, pathways and other physiological phenomena to each other. This is a unique feature in the structure, which allows the annotation of a concept represented in one ontology to a concept represented in a second ontology. A curator may annotate a disease (Alagille Syndrome) to a pathway (Notch Signalling) and indicate the associated mutation in a single record (Jag 1, multiple locations), as illustrated in Figure 3. Ontologies for rat anatomy, cell types, developmental stages, drugs, genetic factors and environmental conditions, as well as qualifiers added to the system, facilitate integration and representation of complex phenotype, disease, expression, pathway and pharmacological data. Phenotype annotations will thus include not only the phenotype ontology term, but also the actual value and the experimental and genetic factors involved. The use of the Experiment/Assay Data Object allows RGD

Copyright © 2006 John Wiley & Sons, Ltd. 

| Strain | Assay Type | Phenotype | Relative to | Value | Diet Condition | Value | Atmosphere Condition | Value | Gender |
|---|---|---|---|---|---|---|---|---|---|
| LE/BluGill | Standard blood chemistry panel | Decreased eosinophil count | PGA avg | 0.0089E3 | Decreased sodium content | 0.4% | Decreased oxygen | 12% | Female |
| FHH/EurMCwi | Jaffe Reaction autoanalyzer | Increased creatinine clearance | PGA avg | 1.3806 ml/min | Increased sodium content | 4.0% | Standard oxygen content | 21% | Female |

| Gene | Disease | Genetic Factor | Environmental Factor | Qualifier |
|---|---|---|---|---|
| Slc6a3 | ADHD | Polymorphism: 3' VNTR | Maternal Smoking | prenatal |

| Pathway | Qualifier | Disease | Genetic Factor | Value |
|---|---|---|---|---|
| Notch Signaling | Altered | Alagille Syndrome | Mutation | Jag1: multiple locations |

| Gene | Expression | BioMaterial | | | | Genetic Factor | Genetic Factor | Relative to | Assay |
|---|---|---|---|---|---|---|---|---|---|
| | | Developmental Stage | Anatomy | Cell Type | Disease | | | | |
| Serpine1 | Increased Expression | Adult:: 12 wks | Abdomen | Brown fat cell | Diabetes Mellitus | Strain:ZDF | Gender: Male | Strain:Z | RT-PCR |

**Figure 3.** Sample records using multiple ontogeny annotations to include pharmacological and physiological data that is not tied to a specific genetic or genomic object. The protocol table is designed to hold additional information required in the MIAME standards for expression data and for storing complete phenotype protocols.

Because of the flexibility of the composite annotation records, data mining tools can be designed to allow researchers to search for data based on any of the ontologies used. The Phenotype Search and Analysis Tool (Figure 4) allows the user to customize datasets through a series of filtering choices presented to the user, based on a previous selection and data availability. Thus, a user selecting a broad trait area is presented with a list of associated phenotypes. Based on the phenotype(s) selected, the user is presented with choices of experimental conditions for which there is data available. Because of the ontological structure of the data for all areas, users have the option to sort data by broad or narrow classifications as they wish. A user can filter phenotype data by all diet conditions, only those with changed mineral content or more narrowly by specific percentage of sodium content. Tools can be designed in a modular fashion to allow users to approach data as they wish. Rather than beginning with a phenotype, a user will be able to begin with an experimental condition, such as high salt content diet, and then be presented with all phenotypes associated with the condition and for which there is data in the database and proceed to filter data by other fields.

RGD has developed an infrastructure to efficiently access ontology annotations in the database for its Quick and Advanced search tools and its GViewer tool. This infrastructure will also be used to query multiple ontology annotations. The method used to query the data is based on PL/SQL functions and procedures in an Oracle 9i database. Matching annotations to terms or descendants of terms are selected and stored in temporary tables in the database. The experimental IDs of these matching annotations are then used to query the database for associated annotations from any other ontology. The iterative querying enables the mining of the

Copyright © 2006 John Wiley & Sons, Ltd.

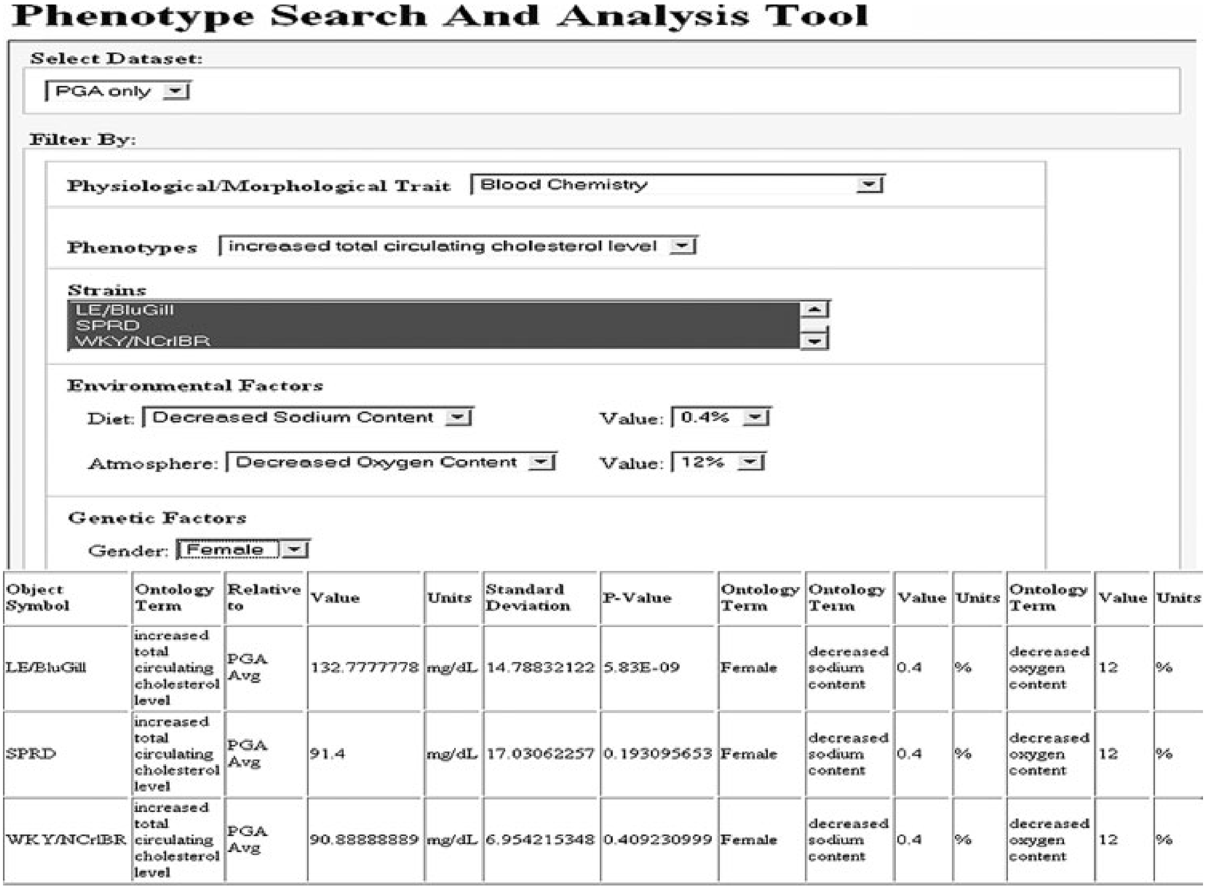

| Object Symbol | Ontology Term | Relative to | Value | Units | Standard Deviation | P-Value | Ontology Term | Ontology Term | Value | Units | Ontology Term | Value | Units |
|---|---|---|---|---|---|---|---|---|---|---|---|---|---|
| LE/BluGill | increased total circulating cholesterol level | PGA Avg | 132.7777778 | mg/dL | 14.78832122 | 5.83E-09 | Female | decreased sodium content | 0.4 | % | decreased oxygen content | 12 | % |
| SPRD | increased total circulating cholesterol level | PGA Avg | 91.4 | mg/dL | 17.03062257 | 0.193095653 | Female | decreased sodium content | 0.4 | % | decreased oxygen content | 12 | % |
| WKY/NCrlBR | increased total circulating cholesterol level | PGA Avg | 90.88888889 | mg/dL | 6.954215348 | 0.409230999 | Female | decreased sodium content | 0.4 | % | decreased oxygen content | 12 | % |

**Figure 4.** Phenotype data-mining tool data by selecting parameters from multiple ontologies that are co-associated. The tools are designed in Java JDK 1.3 and graphical reports use SVG technologies.

### Results and Discussion

Because the rat is used by a diverse community involved in physiological and disease research, investigators are often unsure of the best model to use to study particular phenotypes. By integrating environmental and genetic factors into our model, as well as the inclusion of actual values, RGD can provide phenotype analysis tools to aid the researcher in choosing appropriate models based on the phenotype and conditions of interest. The multiple ontology data structure and annotation system supplies the user with an instant view of the processes, phenotypes, pathway(s) and environmental and genetic factors pertinent to a given disease.

The database infrastructure is already shared by several tools and RGD will integrate the multiple ontology structure in a number of tools, which will facilitate navigation across data for complex queries. The design and implementation of additional sophisticated data-mining tools for experimental data will allow investigators to more easily search for the answer to questions such as: 'Under what conditions is an increase in the severity of a phenotype or a change in the expression of a gene or mutant gene observed?'; 'Are diseases associated with the same pathway different in their manifestations because of differences in the nature of the alterations?'; 'Is, for instance, Notch signalling pathway compromised because the promoters of target gene are mutated, the receptors are not properly modified or because mutations in either receptor or ligand interfere with the normal activity?'; 'Are the manifold malformations (heart, eye, vertebral column) of the Alagille syndrome the result

Copyright © 2006 John Wiley & Sons, Ltd. 

of the various instances of *Jag1* ligand mutations, scattered across the entire gene?'; 'Are individuals affected with CADASIL condition more sensitive to environmental stress because the mutations within the Notch3 receptor irrevocably compromise a three-disulphide bond pattern and, for this matter, its structural integrity?'.

It is precisely the ability to navigate between and link instances of expression, genetic and environmental attributes, where ontology annotations could help researchers unthread the interplay between genes, mutations and environment that underlie complex human diseases.

### References

Ashburner M, Lewis S. 2002. On ontologies for biologists: the Gene Ontology — untangling the web. *Novartis Found Symp* **247**: 66–80; Discussion 80–83, 84–90, 244–252.

Bader GD, Betel D, Hogue CW. 2003. BIND: the Biomolecular Interaction Network Database. *Nucleic Acids Res* **31**(1): 248–250.

Dahlquist KD, Salomonis N, Vranizan K, Lawlor SC, Conklin BR. 2002. GenMAPP, a new tool for viewing and analyzing microarray data on biological pathways. *Nat Genet* **31**(1): 19–20.

Joshi-Tope G, Gillespie M, Vastrik I, *et al*. 2005. Reactome: a knowledgebase of biological pathways. *Nucleic Acids Res* **33**: D428–432.

Kanehisa M. 2002. The KEGG database. *Novartis Found Symp* **247**: 91–101; Discussion 101–103, 119–128, 244–252.

Nelson SJ, Johnston D, Humphreys BL. 2001. Relationships in medical subject headings. In *Relationships in the Organization of Knowledge*, Bean CA, Green R (eds). Kluwer Academic: New York; 171–184.

Smith CL, Goldsmith CA, Eppig JT. 2005. The Mammalian Phenotype Ontology as a tool for annotating, analyzing and comparing phenotypic information. *Genome Biol* **6**(1): R7.

Stevens R, Goble CA, Bechhofer S. 2000. Ontology-based knowledge representation for bioinformatics. *Brief Bioinform* **1**: 398–414.

Copyright © 2006 John Wiley & Sons, Ltd. 
